# Editorial: *Campylobacter*-associated food safety

**DOI:** 10.3389/fmicb.2022.1038128

**Published:** 2022-10-26

**Authors:** Jingbin Zhang, Michael E. Konkel, Greta Gölz, Xiaonan Lu

**Affiliations:** ^1^Department of Food Science and Agricultural Chemistry, Faculty of Agricultural and Environmental Sciences, McGill University, Montreal, QC, Canada; ^2^School of Molecular Biosciences, College of Veterinary Medicine, Washington State University, Pullman, WA, United States; ^3^Institute of Food Safety and Food Hygiene, Freie Universität Berlin, Berlin, Germany

**Keywords:** *Campylobacter*, food safety, poultry, food microbiology, ecology, antibiotic resistance

## Introduction

Campylobacteriosis is an enteric bacterial zoonotic infection caused by members of the *Campylobacter* genus (Kirkpatrick and Tribble, 2011). *C. jejuni* (> 85%) and *C. coli* (5–10%) are the most common species associated with the disease (Patrick et al., 2018). Ingestion of as few as 500 bacteria can cause campylobacteriosis (Robinson, 1981). Although *Campylobacter* typically causes self-limiting human gastroenteritis, it can lead to prolonged post-infectious complications, such as Guillain-Barré syndrome, reactive arthritis, and/or post infectious-irritable bowel syndrome (Rees et al., 1995). The treatment of campylobacteriosis poses significant economic burdens worldwide, resulting in $1.56 billion in healthcare costs in the USA, $80 million in Canada, and €2.4 billion in the European Union per year (Devleesschauwer et al., 2017).

The high prevalence of *Campylobacter* in the agri-food system is likely a major contributing factor to the incidence of campylobacteriosis. Due to its microaerobic nature, *Campylobacter* can colonize the intestinal tract of food-producing animals such as poultry, cattle, sheep, and swine (Hansson et al., 2018). However, it can also survive under aerobic conditions and infect humans through the food supply chain by forming biofilms or entering the viable but non-culturable state (Lv et al., 2019; Ma et al., 2022). The main route of infection has been identified as the consumption of contaminated food commodities, such as unpasteurized dairy products, undercooked poultry meat and/or contaminated water (Silva et al., 2011). Therefore, detection, characterization, and reduction of *Campylobacter* in the agroecosystem are of great importance. This mini-review provides an overview of the current trends in understanding *Campylobacter* and its interaction with the agroecosystem. We first introduce the improved methods to detect *Campylobacter* in various agri-food settings. Then, the prevalence of this microbe in the agri-food system as well as its characteristics are summarized. Finally, novel control strategies of *Campylobacter* are summarized and discussed.

## Improved detection of *Campylobacter* in food-related products

Despite culturing methods are recognized as “golden standard” for traditional detection of *Campylobacter* and are still used as guidelines for regulatory purposes, these methods are time-consuming and labor-intensive. Several molecular- and biomolecular-based methods such as polymerase chain reaction (PCR) (Wegmuller et al., 1993), enzyme-linked immunosorbent assay (Sails et al., 2002), and DNA probe (Maher et al., 2003) have been developed for the detection of *Campylobacter* in a rapid and accurate manner. However, most of these methods require expensive equipment and specialized personnel; therefore, they are unsuitable for onsite and rapid screening of *Campylobacter*. Recently, loop-mediated isothermal amplification (LAMP) and RNA-guided clustered regularly interspaced short palindromic repeats–CRISPR-associated (CRISPR-Cas) methods have been recognized as promising detection methods as they are rapid, sensitive, specific, and practical (Yamazaki et al., 2009; Wang et al., 2020). In this special issue, a LAMP-based system was developed by Kreitlow et al. for simultaneous detection and differentiation of *C. jejuni* and *C. coli* in meat products. After enrichment, the inoculated bacteria (*C. jejuni* and *C. coli*) can be simultaneously detected and differentiated using this system and the result was consistent with both real-time PCR and the standard culture method. In another study in this special issue, Huang et al. utilized the CRISPR-Cas method and developed a CRISPR-Cas12b-based system for the detection of *C. jejuni*. The obtained system could specifically detect *C. jejuni* from chicken samples within 40 min, providing a promising onsite detection method to the poultry industry. In addition to these novel detection methods, traditional culture-based methods are still used in certain circumstances. For example, the procedure of ISO 10272-1:2017 was validated by Hazeleger et al. by determining the level of detection at 50% (LOD_50_, the concentration where the probability of detection is 50%) of five *Campylobacter* strains under different conditions (i.e., different enrichment broths and food matrices). Both food matrices (raw milk, chicken skin, and frozen spinach) and enrichment broths (bolton and preston) had a large influence on the LOD_50_ of *Campylobacter* strains.

## Prevalence and characterization of *Campylobacter*

The pathogenicity of *Campylobacter* depends on virulence factors related to chemotaxis, motility, adhesion, invasion, toxin production, and immune evasion (Lopes et al., 2021). Severe cases of campylobacteriosis need antibiotic treatment (Kirkpatrick and Tribble, 2011). However, overuse and misuse of antibiotics in both the human population and animal production have led to antibiotic resistance for *Campylobacter*, treatment failure, and reoccurrence of the disease. To address the potential risks associated with prevalence, resistance, pathogenicity, genetic diversity, and transmission mode, various genotypic [e.g., multilocus sequence typing (MLST), whole genome sequencing (WGS), etc.] and phenotypic (e.g., antimicrobial susceptibility test, serotyping, etc.) methods have been used to identify and characterize this microbe.

### *Campylobacter* in poultry

In this special issue, Gahamanyi et al. isolated *Campylobacter* from a layer poultry farm in South Korea. They assessed the antimicrobial resistance (AMR) profiles, virulence genes, and genetic diversity of *Campylobacter* isolates Gahamanyi et al. A total of 55 isolates were identified from 153 fecal samples, with *C. jejuni* and *C. coli* being 49 and 6, respectively. The isolates contained various virulence genes (facilitating adhesion, invasion and internalization) and AMR genes (conferring resistance to quinolones and tetracycline). The genetic diversity study discovered 13 genotypes, resulting in three new sequencing types. Another study conducted by Bai et al. in this special issue investigated similar characteristics of *Campylobacter* isolated from the slaughtering line of yellow-feathered broilers in Southern China. Among 1,102 samples, 157 were detected to harbor *Campylobacter*. The highest prevalence of *C. jejuni* and *C. coli* was determined in live chickens (53.6%), followed by carcass samples treated after defeathering (27.5%) and evisceration (18.1%). Resistance- and virulence-associated genes were detected in the isolates and the majority (90.4%) of them were identified to be multidrug-resistant (MDR). Furthermore, pulsed-field gel electrophoresis (PFGE) performed on all the isolates indicated cross-contamination of *Campylobacter* throughout the slaughtering line. Multiple strains of *Campylobacter* can simultaneously infect broiler flocks (Höök et al., 2005), with some strains predominant at different periods. Rawson et al. investigated the dynamics of *Campylobacter* prevalence in response to seasonal variation, species-specificity, bird health, and total colonization prevalence within the chicken flock over a year using multiple Bayesian models Rawson et al. *C. jejuni* occurred more frequently in the summer months, while *C. coli* persisted for longer periods, infecting the most susceptible birds within the flock. *Campylobacter* can acquire resistance genes through horizontal gene transfer (Ma et al., 2021). Guernier-Cambert et al. mimicked the real-world conditions for possible horizontal gene transfer of AMR genes of *Campylobacter* between or within species (*C. coli* or *C. jejuni*). *Campylobacter* isolated from different animal hosts (swine or turkeys) were cultivated either *in vitro* through co-culture experiments or *in vivo* with dual-strains in turkeys. The acquisition of AMR genes was evaluated by WGS of parental and recombinant strains. Four independent transfers of AMR genes occurred during co-culture experiments *in vitro*, while only one appeared during *in vivo* dual-strain cultivation. Therefore, the AMR genes of *Campylobacter* bacteria might be transferred across turkey and swine *via* horizontal gene transfer, leading to the increased AMR of *Campylobacter*.

### *Campylobacter* in cattle

*C. jejuni* serotype HS19 was frequently isolated from patients with Guillain-Barré syndrome; therefore, it is acknowledged as a biomarker of this disease. Interestingly, it was prevalent in Chinese cattle during the epidemiological study conducted by Zang et al. Taking this a step further, the same research group comprehensively investigated both genotype and phenotype of HS19 isolates from cattle. All cattle isolates belonged to clinical high-risk lineage and developed resistance to multiple antibiotics. DNA methylation has been shown to play an important role in the pathogenicity of *C. jejuni*, including motility, adherence, and invasion (Kim et al., 2008). Ghatak et al. investigated the methylome of *C. jejuni* YH002, a MDR strain isolated from retail beef liver. One putative motif belonging to the type II restriction-modification system was discovered in the methylome of *C. jejuni* YH002. By comparing the strain to well-studied *C. jejuni* reference strains (81–176 and NCTC 11168), several non-uniform methylation patterns were observed, indicating the existence of type I and type IV restriction-modification systems. Additional investigations into DNA methylation sites within gene promoters result in the regulation of several virulence genes (i.e., a flagella gene, an RNA polymerase sigma factor, etc.).

### *Campylobacter* in swine

In addition to poultry and cattle, swine is another reservoir of *Campylobacter* bacteria, especially *C. coli* (Mataragas et al., 2008; Di Donato et al., 2020). Therefore, it is of great importance to investigate the *Campylobacter* prevalence and to characterize strains isolated from this reservoir. In this special issue, Guk et al. investigated the prevalence, aerotolerance, quinolone resistance, virulence potential, and MLST genotypes of *C. coli* isolated from different swine groups on farms. The characteristics of *C. coli* isolates were compared to understand their relationship between aerotolerance levels. Among 124 *C. coli* isolates, hyper-aerotolerant (HAT, 13.7%) isolates were present in all swine groups and they encoded for virulence-related genes; therefore, these groups are likely to remain on pig farms and re-infect other pigs. Furthermore, all *C. coli* isolates were resistant to quinolones, with several HAT isolates showing high-level ciprofloxacin resistance. HAT *C. coli* from swine shared MLST genotypes with human isolates at the highest portion; therefore, the resistance may be transmitted to humans *via* the food supply chain due to its aerotolerance.

### *Campylobacter* in humans

To investigate the relative contribution of different *C. coli* isolation sources to human infections in the US, Harrison et al. used core genome MLST and minimal multilocus distance analysis to categorize the core genome of *C. coli* strains isolated from food-producing animals, retail poultry meats, human clinical settings, and environmental sources. Poultry isolates showed the highest likelihood of attribution to human isolates followed by environmental whilst cattle and swine isolates shared less similarity. Although a high prevalence of *C. coli* was observed in swine fecal samples, both methods indicated that swine contributed the least to human infections. Similarly, in another study, Kelley et al. isolated *C. jejuni* from various agricultural and environmental sources in East Tennessee and compared their genomes to those of strains isolated from individuals with campylobacteriosis in the same spatial and temporal environment using WGS Kelley et al. Cattle and chicken isolates shared the highest similarity to those bacteria recovered from humans, indicating the possible transmission route of *C. jejuni* in that region. Another study conducted by Zang et al. investigated the pathogenic characteristics of *C. jejuni* isolated from different ecological sources using capsular polysaccharide genotypes, lipooligosaccharide (LOS) classification, and MLST. A close genetic relatedness was identified between cattle isolates and human pathogenic strains. To identify the possible zoonotic transmission of *C. jejuni* from animals to humans on a dairy farm in Michigan, St. Charles et al. used MLST, WGS and LOS-typing to compare the genome of *C. jejuni* strains isolated from 25 calves, 1 dog, and 1 asymptomatic family member. Two cattle isolates were closely related to the human isolate, indicating possible zoonotic transmission.

### *Campylobacter* in small mammals and other environments

*Campylobacter* is a commensal organism in the gastrointestinal tract of small mammals (Meerburg et al., 2006). However, their genotypes and roles in clinical infections have not been systematically studied. Olkkola et al. collected the intestinal content of small wild mammals from their habitats near pig or cattle farms, isolated *Campylobacter* bacteria, and compared their genomes with those of farm animal and human isolates. Although wild mammal might not be the original source of *Campylobacter* isolates colonizing livestock, they may occasionally carry *Campylobacter* to infect livestock and cause human diseases. Aside from small mammals, the presence of *Campylobacter* in wild birds has been reported (Hald et al., 2016). Turkey is a crucial stopover on migratory bird routes among Europe, Asia, and Africa. *Campylobacter* from clonal contents of wild birds in the hunting areas of Turkey were isolated and characterized by Kürekci et al. High *C. coli* prevalence was determined in Eurasian coot (93%) and all *C. coli* isolates belong to clade II and III. Almost all isolates were sensitive to the tested antibiotics, which may be due to the absence of selective pressure and niche adaption of *Campylobacter* in wild birds.

*Campylobacter* is a microaerobic bacterium, but it is able to survive under aerobic conditions outside the intestinal tract of the host (Ma et al., 2022). Shagieva et al. investigated the survival rate of *C. jejuni*, including waterborne isolates, under different environmental conditions (i.e., low temperature, aerobic and microaerobic conditions). This study demonstrated that water is a significant reservoir of *C. jejuni*, and isolates originating from water could survive under stressful environmental conditions for a prolonged period.

### Strategies combatting *Campylobacter*

In recent years, the increased prevalence of campylobacteriosis and the increase in *Campylobacter* AMR isolates have highlighted the need for novel strategies to control this pathogen (Nastasijevic et al., 2020). *Campylobacter* can be transmitted to humans *via* food-to-human or environment-to-human routes. Therefore, control of campylobacteriosis needs to be focused on mitigating potential reservoirs, including the environment, animals, and humans (Johnson et al., 2017). Based on the characteristics of *Campylobacter*, numerous intervention strategies to inhibit or inactivate this microbe have been tested to reduce its prevalence in food-producing animals. A large variety of probiotics, vaccines, plant-derived compounds, and antimicrobial alternatives (e.g., bacteriophages, bacteriocins, etc.) have been identified as potential candidates to control *Campylobacter* (Riddle and Guerry, 2016; Saint-Cyr et al., 2016; Hakeem and Lu, 2020). In this special issue, two research groups provided reviews on the use of probiotics, primarily lactic acid bacteria, as chicken feed additives in controlling *Campylobacter* infections. Probiotics can reduce the intestinal colonization by pathogens, but this beneficial effect is largely dependent on various factors, such as the type and amount of probiotic bacterial strains used, time and method of administration. In the meanwhile, probiotics need to be co-administrated with other strategies to achieve bacterial elimination Deng et al.; Wyszyńska and Godlewska.

### Vaccine development

To prevent the development of MDR in *C. jejuni*, researchers have developed vaccines and tested other antimicrobial compounds. Cao et al. investigated the pangenome of 173 *C. jejuni* strains and analyzed their virulence factor-related genes. Five core virulence factor proteins with high antigenicity were identified that could be used as the targets of human vaccines. *N*-glycan on *C. jejuni* surface is an invariable carbohydrate structure and it has been demonstrated to be immunogenic in mice, rabbits, and humans (Nothaft and Szymanski, 2010). It was validated to be effective to inhibit *Campylobacter* colonization in chickens by inducing *N*-glycan-specific response (Nothaft et al., 2017). To understand the underlying mechanism, Nothaft et al. inoculated broiler birds with the attenuated *E. coli* live strain expressing superficial *C. jejuni N*-glycan and conducted the vaccination and challenge studies. Genetic differences, microbiota composition, and levels of vaccine-induced IgY in different chicken hosts (responder and non-responder) were compared and analyzed. Level of vaccine-induced IgY as well as the microbial composition of boiler birds affected the effectiveness of *E. coli* vaccine, while genetic difference and serum glycome did not. These information could potentially be used to improve the efficacy of vaccines for *C. jejuni* colonization.

### Bacteriophages

Bacteriophages can specifically infect bacteria and cause the lysis of bacterial cell membranes (Hanlon, 2007). Many researchers have validated the effectiveness of bacteriophages in inhibiting various bacterial pathogens, such as *E. coli, Listeria monocytogenes*, and *Salmonella* (Carlton et al., 2005; Hudson et al., 2013; Huang et al., 2018). However, few studies have focused on the inhibition of *Campylobacter* using bacteriophage, especially at different stages of food production. Steffan et al. screened lytic phages and selected two promising candidates to reduce *C. jejuni* loads under different food processing environmental conditions, such as different temperatures and pH values. Zampara et al. constructed innolysins by fusing endolysin to the phage receptor binding protein of *C. jejuni*. Endolysin can degrade the peptidoglycan in the cell wall of host bacteria, resulting in the release of replicated phages and bacterial inactivation (Loessner, 2005). The developed innolysins exhibited excellent antibacterial activity (1-2 log reduction) against various *C. jejuni* strains both in broth and on the surface of chicken skin. A previous work has demonstrated that bacteria could acquire resistance to defend against phage attack by 1) spontaneous mutations (e.g., phase variation), 2) restriction-modification systems, and 3) adaptive immunity *via* the CRISPR-Cas system (Oechslin, 2018). Phase variation is a strategy that bacteria developed for the adaption to their reservoir. Phase variation of *Campylobacter* during host colonization and infection was reviewed by Cayrou et al. In another study, Sørensen et al. investigated the sensitivity of *C. jejuni* strains isolated from Danish broilers to bacteriophages and identified possible resistance mechanisms. Although over half *C. jejuni* strains were sensitive to at least one phage, several *C. jejuni* strains developed resistance using novel internal resistance mechanisms other than phase variation, restricting the infection of phage to the natural habitat of *C. jejuni*. While bacteriophage represents an exciting alternative to control *Campylobacter*, these publications highlight the need for additional research to understand *C. jejuni* resistance to bacteriophage.

### Targeting biofilms

Biofilm formation regulated by quorum sensing (QS) is critical for the survival of *C. jejuni* and its high prevalence in the environment (Ma et al., 2022). Many phytochemicals, fatty acids, and peptides have been studied for *C. jejuni* intervention. To develop alternative strategies for the control of *C. jejuni* in the agroecosystem, Li et al. investigated and validated the effectiveness of two fatty acids (i.e., decanoic acid and lauric acid) in modulating QS signal and biofilm formation of tested *C. jejuni* strains. Similarly, Talukdar et al. used puroindolines to inhibit the growth and biofilm formation of *C. jejuni*. Besides the inhibitory effect of the antimicrobials, their mechanisms of action were investigated. Wagle et al. applied various phytochemicals to chicken skin that were artificially inoculated with *C. jejuni* and tested their effectiveness in bacterial inhibition. Subinhibitory concentration for adhesion, quorum sensing, and gene expression analyses were used to uncover the inhibition mechanisms. The tested phytochemicals reduced *C. jejuni* counts on chicken skin and exhibited their antimicrobial capability by reducing the adherence, inhibiting quorum sensing activity, and disrupting the cell wall structure of *Campylobacter*. Therefore, biofilm-associated metabolism could be used as potential target for various antimicrobial compounds.

## Conclusion

*Campylobacter* in the agroecosystem generates a great concern in the food industry and public health, calling for a better understanding of this microbe and its interaction with the agri-food system, the development of rapid and accurate detection methods as well as effective intervention strategies. With the development of molecular techniques, various identification, detection, typing, and sequencing methods have been applied to understand the characteristics of *Campylobacter*. The underlying mechanisms of its prevalence in the environment (i.e., survival, ecology, adaptation, virulence, and antimicrobial resistance) have also been elucidated. Furthermore, various novel control strategies have been developed for the control of *Campylobacter* in agri-food products. In conclusion, the articles in this special issue contributed to developing systemic approaches to improving the *Campylobacter*-associated food safety.

## Author contributions

JZ leads the writing of this editorial summary. MK, GG, and XL contributed to review, edit, and comment. All authors contributed to the article and approved the submitted version.

## Funding

Note that Frontiers in Microbiology office has agreed to waive the payment of this publication as it is the editorial summary of the special issue *Campylobacter*-associated food safety (https://www.frontiersin.org/research-topics/14193/campylobacter-associated-food-safety). As the topic editors, we were invited by Frontiers in Microbiology to draft this editorial summary.

## Conflict of interest

The authors declare that the research was conducted in the absence of any commercial or financial relationships that could be construed as a potential conflict of interest.

## Publisher's note

All claims expressed in this article are solely those of the authors and do not necessarily represent those of their affiliated organizations, or those of the publisher, the editors and the reviewers. Any product that may be evaluated in this article, or claim that may be made by its manufacturer, is not guaranteed or endorsed by the publisher.
