# Supplementary material for: Editorial: Campylobacter-associated food safety
Source: Front Microbiol. 2022 Oct 26;13:1038128. doi: 10.3389/fmicb.2022.1038128 (PMC9644199; doi:10.3389/fmicb.2022.1038128)
Supplement: Supplementary file 1 [file Table_1.DOCX]

| **Study** | **Topics** | **Main findings** |
| --- | --- | --- |
| Kreitlow et al., 2021 | Detection and differentiation of *C. jejuni* and *C. coli* in meat products using LAMP-based assays | *C. jejuni* and *C. coli* were simultaneously detected and differentiated in meat products. |
| Huang et al., 2021 | Development of a CRISPR-Cas12b-based system for the detection of *C. jejuni* | *C. jejuni* could be detected from chicken samples with a limit of detection (LOD) of 10 CFU/g in 40 min. |
| Hazeleger et al., 2022 | Determination of the LOD_50_ of *Campylobacter* spp. under different conditions using the culture-based method | Both food matrices (raw milk, chicken skin, and frozen spinach) and enrichment broths (bolton and preston) affected the LOD_50_ of *Campylobacter* strains. |
| Gahamanyi et al., 2021 | Antimicrobial resistance profiles, virulence genes, and genetic diversity of *Campylobacter* spp. isolated from a layer poultry farm in Korea | 1) *Campylobacter* isolates contained various virulence genes and antimicrobial resistance (AMR) genes; 2) three new sequencing types were discovered. |
| Bai et al., 2021 | Prevalence, antimicrobial resistance, virulence genes, and genetic diversity of *Campylobacter* spp. along the yellow-feathered broiler slaughtering line in Southern China | 1) Highest prevalence of *C. jejuni* and *C. coli* was determined in live chickens, followed by carcass samples treated after defeathering and evisceration; 2) resistance- and virulence-associated genes were detected in the isolates and the majority (90.4%) of them were identified to be multidrug-resistant. |
| Rawson et al., 2020 | Investigating the dynamics of *Campylobacter* spp. prevalence within a chicken flock over a year using multiple Bayesian models | *C. jejuni* occurred more frequently in the summer months, while *C. coli* persisted for longer periods, infecting the most susceptible birds within the flock. |
| Guernier-Cambert et al., 2021 | Natural horizontal gene transfer of AMR genes in *Campylobacter* spp. from turkeys and swine | AMR genes of *Campylobacter* bacteria might be transferred across turkey and swine by horizontal gene transfer. |
| Zang et al., 2021a | Investigating the genomic relatedness, antibiotic resistance, and virulence traits of *C. jejuni* HS19 isolates from cattle in China | All cattle isolates belonged to clinical high-risk lineage and developed resistance to multiple antibiotics. |
| Ghatak et al., 2020 | Comparative methylome analysis of *C. jejuni* strain YH002 | 1) Existence of type I and type IV restriction-modification systems were found; 2) DNA methylation sites within gene promoters result in regulation of several virulence genes (i.e., a flagella gene, an RNA polymerase sigma factor, etc.) |
| Guk et al., 2021 | Investigating the prevalence, aerotolerance, virulence potential, and MLST genotypes of *C. coli* isolated from different swine groups on farms | 1) Hyper-aerotolerant isolates were present in all swine groups and they encoded virulence-related genes; 2) the resistance of *C. coli* isolates might be transmitted to humans due to its aerotolerance. |
| Harrison et al., 2021 | Categorization of the core genome of *C. coli* strains isolated from various agri-food settings using core genome MLST and minimal multilocus distance analysis | Poultry isolates showed the highest likelihood of attribution to human clinical isolates. |
| Kelley et al., 2020 | Whole-genome sequencing and bioinformatic analysis of environmental, agricultural, and human C. *jejuni* isolates from East Tennessee | Cattle and chicken isolates shared the highest similarity to those bacteria recovered from humans. |
| Zang et al., 2021b | Characterization of *C. jejuni* isolated from different ecological sources using capsular polysaccharide genotypes, lipooligosaccharide (LOS) classification, and MLST | Cattle isolates had a close genetic relatedness with human pathogenic strains. |
| St Charles et al., 2022 | Identification of the possible zoonotic transmission of *C. jejuni* from animals to humans on a dairy farm | Two cattle isolates were closely related to the human isolate, indicating possible zoonotic transmission. |
| Olkkola et al., 2020 | Host-dependent clustering of *Campylobacter* strains from small mammals in Finland | The wild mammal may occasionally carry *Campylobacter* to infect livestock and cause human diseases. |
| Kürekci et al., 2021 | Characterization of *Campylobacter* spp. strains isolated from wild birds in Turkey | High *C. coli* prevalence was determined in Eurasian coot (93%) and all *C. coli* isolates belong to clade II and III. |
| Shagieva et al., 2021 | Investigating the survival rate of *C. jejuni* under different environmental conditions | *C. jejuni* isolates originating from water could survive under stressful environmental conditions for a prolonged period. |
| Deng et al., 2020 | Current perspectives and potential of probiotics to limit *Campylobacter* in poultry | Probiotics is effective in reducing *Campylobacter* in poultry, but it needs to be co-administrated with other strategies to achieve elimination. |
| Wyszyńska and Godlewska, 2021 | The effects of *Campylobacter* infection on the chicken microbiome and colonization control strategies using probiotics | Probiotics can reduce the intestinal colonization by pathogens, but this beneficial effect is largely dependent on various factors (e.g., the type and amount of probiotic bacterial strains used, the time and method of administration). |
| Cao et al., 2021 | Identification of the potential core vaccine targets for *C. jejuni* using multi-omics | Five core virulence factor proteins with high antigenicity were identified as the targets of human vaccines. |
| Nothaft et al., 2021 | Improving chicken responses to glycoconjugate vaccination against *C. jejuni* | The level of vaccine-induced IgY as well as the microbial composition of boiler birds affected the effectiveness of *E. coli* vaccine, while genetic difference and serum glycome did not. |
| Steffan et al., 2021 | Isolation and characterization of group III *C. jejuni*-specific bacteriophages from Germany and their suitability for use in food production | Two lytic phages reduced *C. jejuni* loads under different food processing settings. |
| Zampara et al., 2021 | Developing innolysins against *C. jejuni* using a novel prophage receptor-binding protein | The developed innolysins exhibited excellent antibacterial activity against various *C. jejuni* strains both in broth and on the surface of chicken skin. |
| Cayrou et al., 2021 | Phase variation during host colonization and invasion by *Campylobacter* spp. | 1) PV Genes *cj0170* and *cj0045* are strongly associated with host colonization; 2) Individual PV genes have different functions on different *Campylobacter* spp. during colonization. |
| Sørensen et al., 2021 | Identification of novel phage resistance mechanisms in *C. jejuni* by comparative genomics | Although over half *C. jejuni* strains were sensitive to at least one phage, several *C. jejuni* strains developed resistance using novel internal resistance mechanisms other than phase variation. |
| Li et al., 2021 | Inhibition of AI-2 quorum sensing and biofilm formation in C. *jejuni* by decanoic and lauric acids | Decanoic acid and lauric acid were effective in modulating QS signal and biofilm formation of tested *C. jejuni* strains. |
| Talukdar et al., 2021 | Inhibitory effect of puroindoline peptides on *C. jejuni* growth and biofilm formation | PinA has a strong antimicrobial effect against *C. jejuni* and its biofilm formation. |
| Wagle et al., 2021 | Investigating the antimicrobial effect of phytochemicals in reducing *C. jejuni* in postharvest poultry and modulating the virulence attributes of *C. jejuni* | The tested phytochemicals reduced *C. jejuni* counts on chicken skin and exhibited their antimicrobial capability by reducing the adherence, inhibiting quorum sensing activity, and disrupting the cell wall structure of *Campylobacter*. |
